# Targeting USP22 to promote K63-linked ubiquitination and degradation of SARS-CoV-2 nucleocapsid protein

**DOI:** 10.1128/jvi.02234-24

**Published:** 2025-04-04

**Authors:** Xin Xiao, Shifeng Li, Zhijin Zheng, Yingying Ji, Qian Du, Yibo Zuo, Ying Miao, Yukang Yuan, Hui Zheng, Fang Huang, Jun Wang

**Affiliations:** 1 Department of Critical Care Medicine, The First Affiliated Hospital of Soochow University, Suzhou, China; 2 International Institute of Infection and Immunity, Institutes of Biology and Medical Sciences (IBMS), Collaborative Innovation Center of Hematology, MOE Key Laboratory of Geriatric Disease and Immunology of Ministry of Education of China, School of Medicine, Soochow University, Suzhou, Jiangsu, China.; 3 Department of Laboratory Medicine, Institute of Laboratory Medicine, Sichuan Provincial People's Hospital, School of Medicine, University of Electronic Science and Technology of China 12599, Chengdu, Sichuan, China; The Ohio State University, Columbus, Ohio, USA

**Keywords:** deubiquitination, SARS-CoV-2 NP, sulbactam, USP22, viral infection

## Abstract

Severe acute respiratory syndrome coronavirus 2 (SARS-CoV-2) generally hijacks the cellular machinery of host cells for survival. However, how SARS-CoV-2 employs the host’s deubiquitinase to facilitate virus replication remains largely unknown. In this study, we identified the host deubiquitinase USP22 as a crucial regulator of the expression of SARS-CoV-2 nucleocapsid protein (SARS-CoV-2 NP), which is essential for SARS-CoV-2 replication. We demonstrated that SARS-CoV-2 NP proteins undergo ubiquitination-dependent degradation in host cells, while USP22 interacts with SARS-CoV-2 NP and downregulates K63-linked polyubiquitination of SARS-CoV-2 NP, thereby protecting SARS-CoV-2 NP from degradation. Importantly, we further revealed that sulbactam, an antibiotic, can reduce USP22 protein levels, eventually promoting the degradation of SARS-CoV-2 NP *in vitro* and *in vivo*. This study reveals the mechanism by which SARS-CoV-2-encoded NP protein employs host deubiquitinase for virus survival and provides a potential strategy to fight against SARS-CoV-2 infection.

**IMPORTANCE:**

Severe acute respiratory syndrome coronavirus 2 (SARS-CoV-2) nucleocapsid protein (SARS-CoV-2 NP) plays a pivotal role in viral infection by binding to viral RNA, stabilizing the viral genome, and promoting replication. However, the interactions between SARS-CoV-2 NP and host intracellular proteins had not been elucidated. In this study, we provide evidence that SARS-CoV-2 NP interacts with the deubiquitinase USP22 in host cells, which downregulates SARS-CoV-2 NP ubiquitination. This reduction in ubiquitination effectively prevents intracellular degradation of SARS-CoV-2 NP, thereby enhancing its stability, marking USP22 as a potential target for antiviral strategies. Additionally, our findings indicate that sulbactam significantly decreases the protein levels of USP22, thereby reducing SARS-CoV-2 NP levels. This discovery suggests a novel therapeutic pathway in which sulbactam could be repurposed as an antiviral agent, demonstrating how certain antibiotics might contribute to antiviral treatment. This work thus opens avenues for drug repurposing and highlights the therapeutic potential of targeting host pathways to inhibit viral replication.

## INTRODUCTION

Severe acute respiratory syndrome coronavirus 2 (SARS-CoV-2), the pathogen responsible for the ongoing coronavirus disease-2019 (COVID-19) pandemic, has had an unprecedented impact on global social and economic development. More critically, it has created a significant public health crisis worldwide. Multiple open reading frames in the SARS-CoV-2 genome encode 4 structural proteins (1), 16 non-structural proteins, and other accessory proteins (2). These structural proteins include the spike glycoprotein (S), envelope protein (E), matrix protein (M), and nucleocapsid protein (N; SARS-CoV-2 NP). Of all the coronaviruses, the nucleocapsid protein is the most abundantly expressed structural protein during the infection (3). During SARS-CoV-2 infection, SARS-CoV-2 NP binds to the viral RNA genome, interacting with M protein to facilitate transcription efficiency and contribute to virus replication (4). Moreover, SARS-CoV-2 NP has been reported to enhance NF-κB activation, thereby increasing inflammation (5). Several studies have shown that SARS-CoV-2 NP suppresses RNA virus-induced type I interferon (IFN) production (6–8), aiding the virus in evading the immune response. In addition, the severity of the disease is correlated with antibodies against SARS-CoV-2 NP (9). Given these factors, SARS-CoV-2 NP is an ideal diagnostic and treatment target.

Ubiquitin-specific peptidase 22 (USP22) is a member of the ubiquitin-specific processing proteases (USPs) and is recognized as a biomarker for predicting tumor metastasis and recurrence due to its overexpression in malignant tumors (10, 11). During virus infection, USP22 interacts with USP13 and specifically cleaves K27-linked ubiquitin chains on STING (12). Concurrently, USP22 promotes the antiviral response by stabilizing KPNA2 and facilitating IRF3 nuclear translocation (13). The activation of STING activates the downstream transcription factors IRF3 and NF-κB by interacting with proteins such as TANK-binding kinase 1 (TBK1) and IRF3, while phosphorylation of IRF3 promotes the production of interferon type I (IFN-I), a key factor in anti-viral immunity. To date, no research has investigated the relationship between USP22 and viral proteins. A recent study has reported vitamin C (Vit-C)-mediated regulation of ubiquitination on SARS-CoV-2 infection, which is involved in ubiquitination and degradation of ACE2 (14). Additionally, SARS-CoV-2 has been shown to increase the expression of USP5, which inhibits type I IFN signaling, thereby enhancing viral replication (15). It is well established that post-translational modifications (PTMs) of proteins significantly influence coronavirus infection and replication *in vivo* (16). In this study, we unveiled a clear interaction between SARS-CoV-2-encoded NP and the host’s deubiquitinase USP22, providing new insights into SARS-CoV-2 prevention.

Sulbactam, a competitive and irreversible beta-lactamase inhibitor (17), primarily targets bacterial species such as *Neisseria gonorrhoeae* and *Bacteroides fragilis*. Recently, it has been combined with cephalosporins and carbapenems to restore antibiotic efficacy and maintain sensitivity (18). Interestingly, through our screening, we discovered that sulbactam may also exhibit antiviral effects by decreasing USP22 protein levels in a dose- and time-dependent manner. Our further studies demonstrated that sulbactam downregulates SARS-CoV-2 NP, protecting against SARS-CoV-2 infection. This intriguing finding provides new insights into the potential role of antibiotic therapy in viral infections.

## RESULTS

### Identification of USP22 as the deubiquitinase for SARS-CoV-2 NP regulation

**Fig 1:**
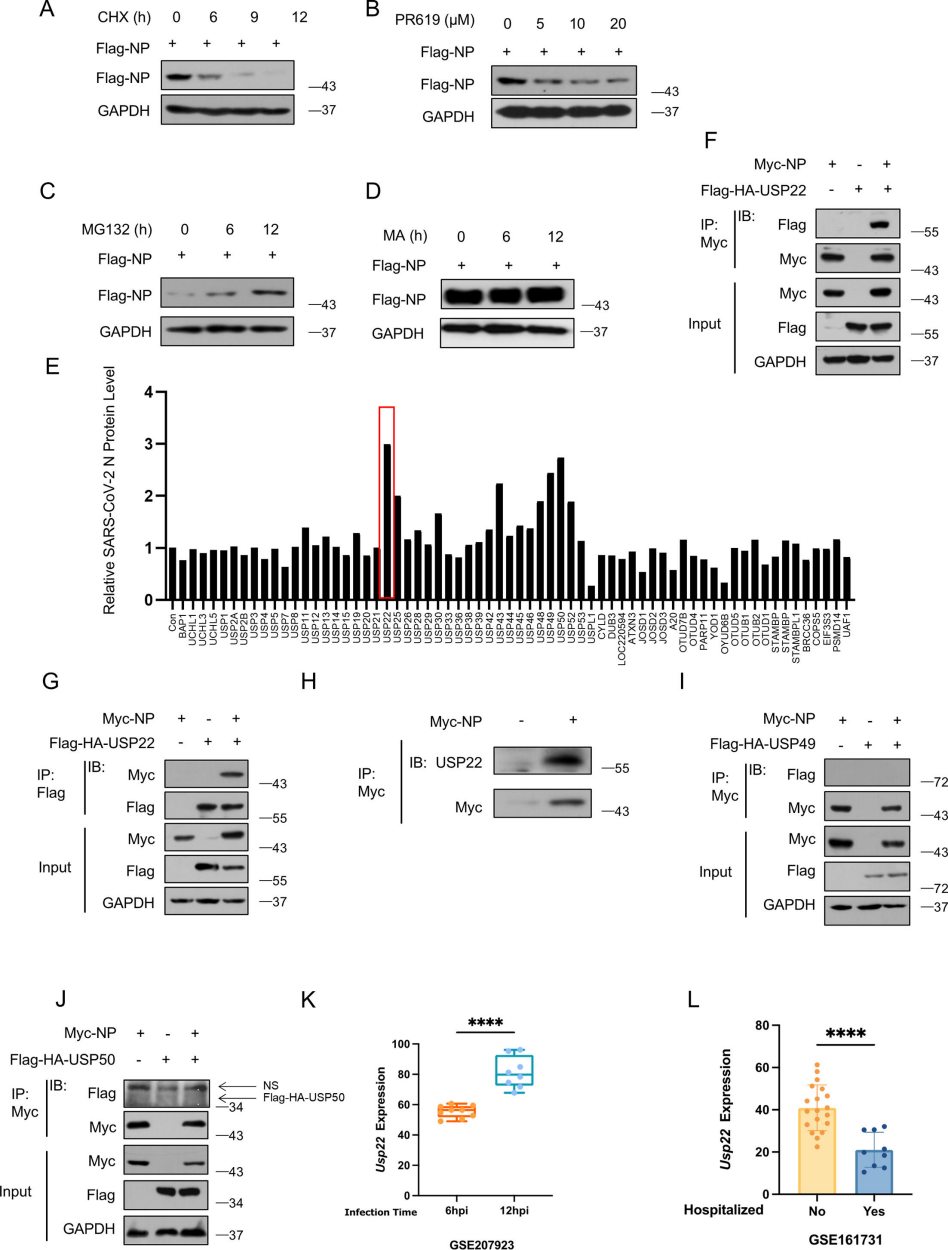
The deubiquitinase interacts with SARS-CoV-2 NP. (**A**) Western blot analysis of Flag-NP in HEK293T cells transfected with Flag-NP and then treated with CHX (50 µM) for 6, 9, and 12 h. (**B**) Western blot analysis of Flag-NP in HEK293T cells transfected with Flag-NP and then treated with PR619 (0, 5, 10, and 20 µM, 2 h). (**C**) Western blot analysis of Flag-NP in HEK293T cells transfected with Flag-NP and then treated with MG132 (10  µM) for 6 and 12 h. (**D**) Western blot analysis of Flag-NP in HEK293T cells transfected with Flag-SARS-CoV-2-N and then treated with MA (10  µM) for 0, 6, and 12 h. (**E**) HEK293T cells were individually transfected with the plasmids from the human deubiquitinating enzyme (DUB) expression library. Western blot was used to identify the key deubiquitinase that significantly increases Flag-NP levels (the intensity of Flag-NP/GAPDH bands measured by Image J) normalized to the empty vector group (CON). (**F–I**) Immunoprecipitation-Immunoblotting (IP-IB) analysis of the interaction between Myc-NP and possible deubiquitinase (Flag-HA-USP22, Flag-HA-USP49, and Flag-HA-USP50) in HEK293T cells cotransfected with these two plasmids. (**J**) IP-IB analysis of the interaction between Myc-NP and endogenous USP22 in HEK293T cells. (**K and L**) Differential *Usp22* expression analysis of GSE207923 data and GSE161713 data samples. ****P < 0.0001, two-tailed unpaired Student’s *t*-test and Mann-Whitney test (**H**). Data are representative of three biological replicates (**A–J**).

To investigate the degradation mechanisms of SARS-CoV-2 NP, we first used cycloheximide (CHX), a protein biosynthesis inhibitor, to assess the stability of Flag-NP. We observed a gradual decrease in protein levels as time increased, indicating that SARS-CoV-2 NP could degrade in the cells (Fig. 1A). Next, we treated the cells with PR-619, a non-selective deubiquitinating enzyme inhibitor, and found that Flag-NP decreased over time, suggesting that deubiquitinating enzymes may affect SARS-CoV-2 NP (Fig. 1B). Also, we observed an increase in Flag-NP levels following the addition of the proteasome inhibitor MG132 (Fig. 1C), whereas Flag-NP levels decreased after being treated with the lysosomal inhibitor methyladenine (MA; Fig. 1D), indicating that SARS-CoV2 NP is primarily degraded through the ubiquitin-proteasomal pathway. Subsequently, we used a deubiquitinase expression library to identify the potential deubiquitinase. We found that despite the potential involvement of multiple deubiquitinases in the regulation of SARS-CoV-2 NP levels, the deubiquitinase USP22 showed the most significant effect on SARS-CoV-2 NP upregulation, along with USP49 and USP50 (Fig. 1E). Further immunoprecipitation (IP) analysis revealed a clear interaction between Flag-HA-USP22 and Myc-NP, regardless of the immunoprecipitation of Myc-NP (Fig. 1F) or Flag-HA-USP22 (Fig. 1G). Additionally, there was a constitutive interaction between endogenous USP22 and SARS-CoV-2 NP (Fig. 1H). In contrast, USP49 and USP50 did not interact with SARS-CoV-2 NP (Fig. 1I and J). To further investigate the role of USP22 in the SARS-CoV-2 infection process, we analyzed the disparity of *Usp22* between groups from the Gene Expression Omnibus database. We observed a significant increase in *Usp22* expression associated with the duration of SARS-CoV-2 infection of normal human bronchial epithelial cells from data sets GSE207923 (19) (Fig. 1K). Conversely, in data set GSE161731, patients who required hospitalization exhibited lower *Usp22* expression than those who did not require hospitalization (20) (Fig. 1L). In summary, these findings indicated that the host deubiquitinase USP22 can interact with SARS-CoV-2-encoded NP proteins.

### USP22 upregulates SARS-CoV-2 NP protein levels via its deubiquitinase activity

**Fig 2:**
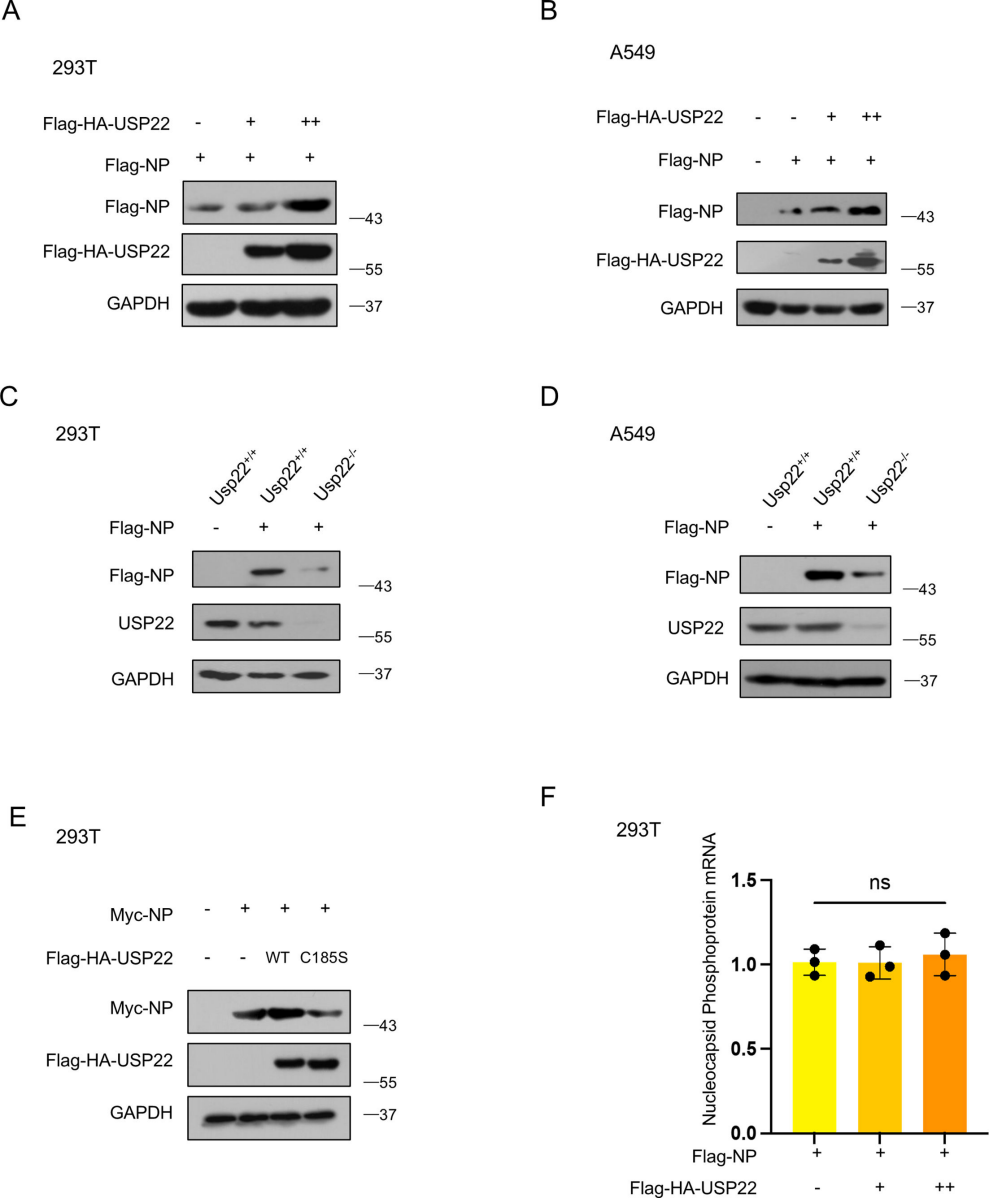
USP22 upregulates SARS-CoV-2 NP. (**A and B**) Western blot analysis of Flag-NP in HEK293T cells and A549 cells transfected with Flag-NP and an increasing amount of Flag-HA-USP22. (**C and D**) Western blot analysis of Flag-NP in *Usp22*^+/+^and *Usp22*^−/−^ HEK293T cells and A549 cells transfected with Flag-NP. (**E**) Western blot analysis of Myc-NP in HEK293T cells cotransfected with Myc-NP and Flag-HA-USP22 or Flag-HA-USP22 (C185S). (**F**) RT-qPCR analysis of SARS-CoV-2 nucleocapsid phosphoprotein mRNA in HEK293T cells transfected with Flag-NP and dose-increased Flag-HA-USP22. ns, not significant (two-tailed unpaired Student’s *t*-test). Data are shown as mean and SD of three biological replicates (**F**) or are representative of three biological replicates (**A–E**).

To investigate the regulatory effect of USP22 on SARS-CoV-2 NP, we first overexpressed USP22 in cells. Our results demonstrated that USP22 overexpression increased the expression of Flag-NP in a dose-dependent manner in HEK-293T cells (Fig. 2A). Similar results were observed in A549 cells (Fig. 2B). Then, we generated USP22‐knockout (KO) cell lines using CRISPR‐Cas9. Interestingly, we found that Flag-NP levels significantly decreased in *Usp22^−/−^* HEK-293T cells (Fig. 2C). The same results were found in *Usp22^−/−^* A549 cells (Fig. 2D). Previous reports have shown that USP22 possesses a deubiquitinating enzyme activity, whereas the USP22 (C185S) mutant lacks this activity (21). Based on this information, we mutated the C185 site to an inactive S (C185S) and examined the regulatory effect of wild-type USP22 (USP22-WT) and its mutant (USP22-C185S) on SARS-CoV2 NP. The results showed that USP22-C185S lost the ability to increase SARS-CoV-2 NP levels (Fig. 2E). Next, we sought to determine whether USP22 affects SARS-CoV-2 NP at the mRNA level. Real-time quantitative PCR (RT-qPCR) analysis revealed that USP22 overexpression did not affect the mRNA level of SARS-CoV-2 NP (Fig. 2F). Taken together, these findings indicated that USP22 upregulates SARS-CoV-2 NP protein levels dependent on its deubiquitinase activity.

### USP22 regulates K63‐linked polyubiquitination of SARS-CoV-2 NP

**Fig 3:**
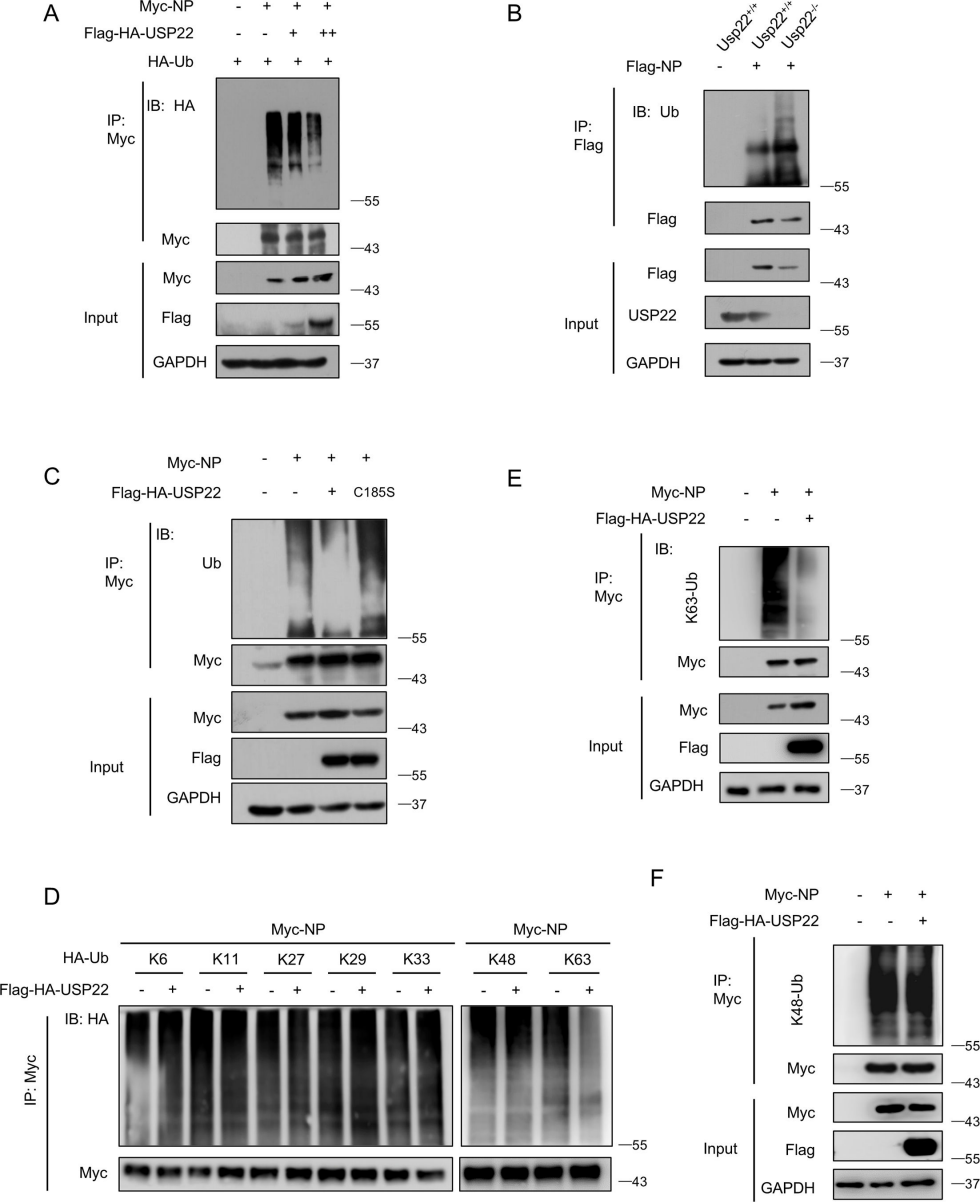
USP22 regulates K63‐linked polyubiquitination of SARS-CoV-2 NP. (**A**) IP-IB analysis of ubiquitination of Myc-NP in HEK293T cells cotransfected with Myc-NP, HA-Ub, and increasing amounts of Flag-HA-USP22. (**B**) IP-IB analysis of ubiquitination of Flag-NP in *Usp22*^+/+^ and *Usp22*^−/−^ HEK293T cells transfected with Flag-NP. (**C**) IP-IB analysis of ubiquitination of Myc-NP in HEK293T cells cotransfected with Myc-NP and Flag-HA-USP22 or Flag-HA-USP22 (C185S). (**D**) IP-IB analysis of ubiquitination types of Myc-NP in HEK293T cells cotransfected with Myc-NP and different types of HA-Ub and then transfected with or without Flag-HA-USP22. The red dashed contour represents the group of K63-linked ubiquitination we focused on studying here. (**E**) IP-IB analysis of K63-linked polyubiquitination (**K63-UB**) of Myc-NP in HEK293T cells transfected with Myc-NP and Flag-HA-USP22, using a specific anti-K63-Ub antibody. (**F**) IP-IB analysis of K48-linked polyubiquitination (**K48-UB**) of Myc-NP in HEK293T cells transfected with Myc-NP and Flag-HA-USP22, using a specific anti-K48-Ub antibody. Data are representative of three biological replicates (**A–F**).

We next elucidated the detailed mechanisms by which USP22 regulates SARS-CoV-2 NP. Our results exhibited that overexpression of USP22 markedly decreased the ubiquitination of Myc-NP (Fig. 3A). Next, we detected the ubiquitination of Myc-NP in *Usp22^−/−^* cells. Consistent with our previous findings, the ubiquitination of Myc-NP was significantly increased in the absence of USP22, accompanied by a reduction in SARS-CoV-2 NP at the protein level (Fig. 3B). Moreover, the ubiquitination level of SARS-CoV-2 NP was not reduced by the deubiquitinase‐inactive mutant of USP22 (USP22C185S) (Fig. 3C). Further analysis of Myc-NP ubiquitination types revealed that USP22 mainly reduced K63-linked polyubiquitination compared to other types of ubiquitination linkage (Fig. 3D). Additionally, we confirmed that USP22 downregulates K63-linked polyubiquitination of SARS-CoV-2 NP by using an anti-K63-linked ubiquitin antibody (Fig. 3E). Meanwhile, we confirmed that USP22 downregulates K63-linked polyubiquitination of SARS-CoV-2 NP using an anti-K63-linked ubiquitin antibody (Fig. 3E). In contrast, no significant effect of USP22 on K48-linked ubiquitination levels of SARS-CoV-2 NP was observed with the anti-K48-linked ubiquitin antibody (Fig. 3F). In summary, the above results suggested that USP22 inhibits the degradation of SARS-CoV-2 NP by downregulating its K63-linked polyubiquitination.

### USP22 stabilizes SARS-CoV-2 NP proteins

**Fig 4:**
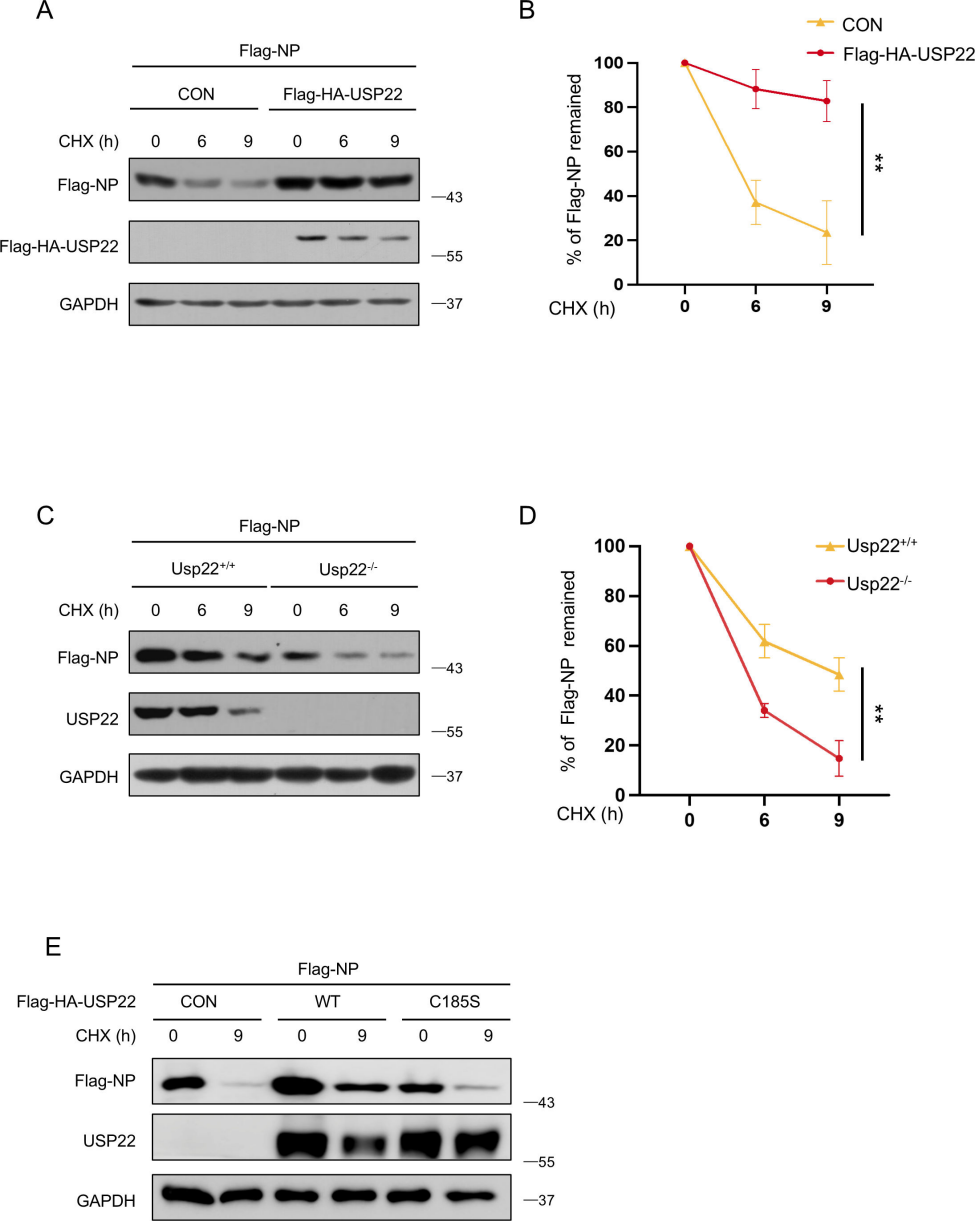
USP22 stabilizes SARS-CoV-2 NP. (**A**) Western blot analysis of Flag-NP in HEK293T cells transfected with Flag-NP and then treated with CHX (50 µg/mL) for the indicated durations. (**B**) The striped images of Flag-NP and GAPDH from (**A**) were imported into Image J. The pixel count of all the strips was measured, and the percentage of Flag-NP that remained was calculated in GraphPad Prism software. (**C**) Western blot analysis of Flag-NP in *Usp22*^+/+^ and *Usp22*^−/−^ HEK293T cells transfected with Flag-NP and then treated with CHX (50 µg/mL) for the indicated durations. (**D**) The striped images of Flag-NP and GAPDH from (**C**) were imported into Image J. The pixel count of all the strips was measured, and the percentage of Flag-NP that remained was calculated in GraphPad Prism software. (**E**) Western blot analysis of Flag-NP in HEK293T cells transfected with Flag-HA-USP22 or Flag-HA-USP22 (C185S) and then treated with CHX (50 µg/mL) for the indicated durations. **P < 0.01 (two-tailed unpaired Student’s *t*-test). Data are shown as mean and SD of three biological replicates (**B and D**) or are representative of three independent experiments (A, C, and E).

Given that USP22 reduced the ubiquitination level of SARS-CoV-2 NP and downregulated its protein levels, we questioned whether USP22 could regulate the stability of SARS-CoV-2 NP. Cycloheximide, a widely used inhibitor of protein synthesis, was employed in a pulse-chase assay (22). Our study showed that overexpression of USP22 significantly decreased the degradation of SARS-CoV-2 NP (Fig. 4A and B). In contrast, in the *Usp22^−/−^* cells, USP22 deficiency significantly accelerated SARS-CoV-2 NP degradation (Fig. 4C and D). Consistent with our study, the CHX pulse-chase assay demonstrated that the deubiquitinase-inactive mutant USP22-C185S failed to stabilize SARS-CoV-2 NP (Fig. 4E). Collectively, these results indicated that USP22 enhances SARS-CoV-2 NP protein stability.

### Sulbactam affects SARS-CoV-2 NP levels by regulating USP22

**Fig 5:**
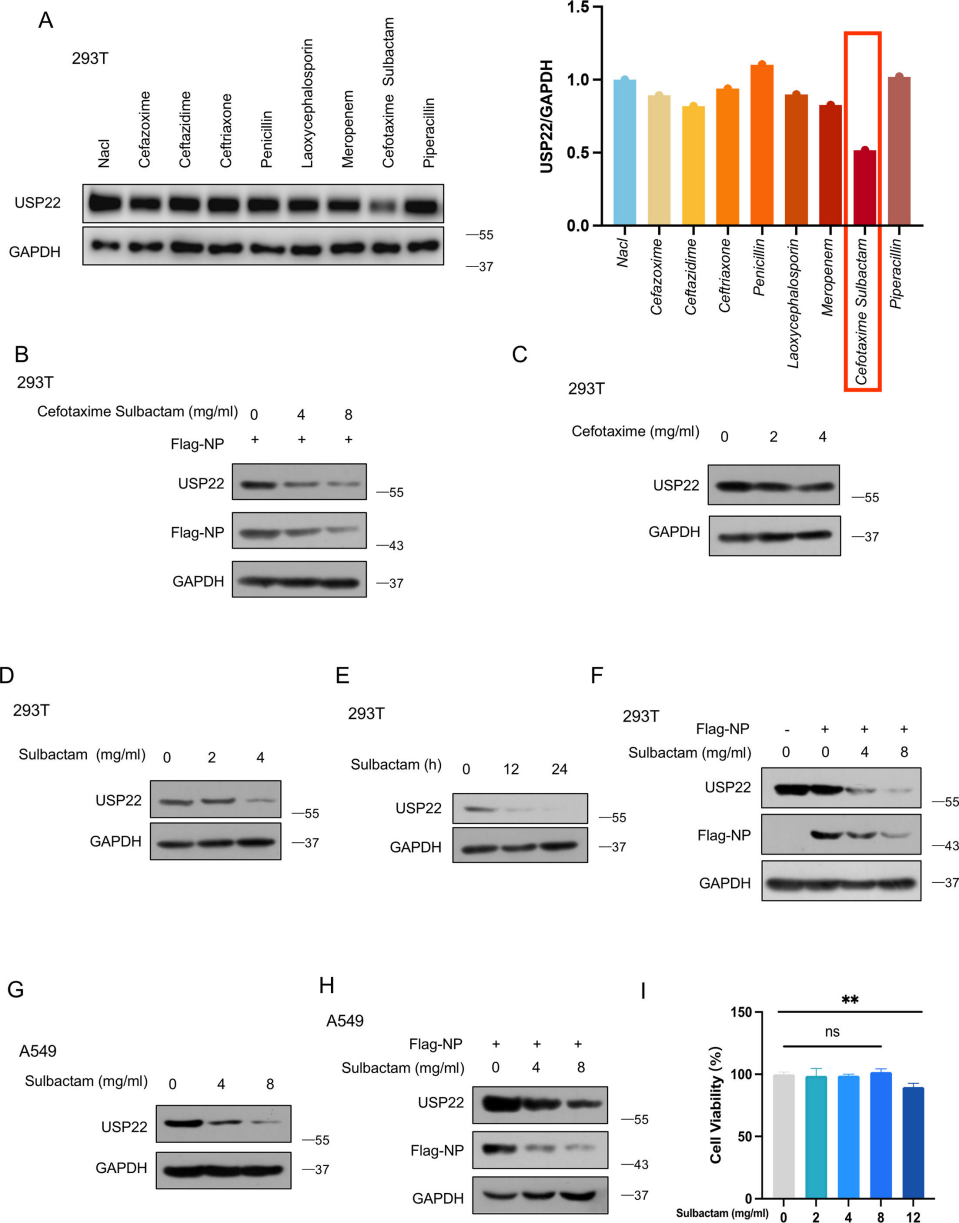
Sulbactam affects SARS-CoV-2 NP levels by regulating USP22. (**A**) Western blot analysis of USP22 in HEK293T cells treated with the indicated drugs (4 mg/mL) for 12 h. (**B**) Western blot analysis of Flag-NP and USP22 in HEK293T cells transfected with Flag-NP and then treated with cefotaxime sulbactam (0, 4, and 8 mg/mL) for the indicated durations. (**C**) Western blot analysis of USP22 in HEK293T cells treated with indicated amounts of Cefotaxime for 12 h. (**D**) Western blot analysis of USP22 in HEK293T cells treated with indicated amounts of sulbactam for 12 h. (**E**) Western blot analysis of USP22 in HEK293T cells treated with sulbactam (4 mg/mL) for the indicated durations. (**F**) Western blot analysis of Flag-NP and USP22 in HEK293T cells transfected with Flag-NP and treated with indicated amounts of sulbactam for 12 h. (**G**) Western blot analysis of USP22 in A549 cells treated with indicated amounts of sulbactam for 12 h. (**H**) Western blot analysis of Flag-NP and USP22 in A549 cells transfected with Flag-NP and treated with indicated amounts of sulbactam for 12 h. (**I**) CCK8 analysis of the effect of sulbactam on the cell viability of HEK293T cells. ns, not significant, **P < 0.01 (two-tailed unpaired Student’s *t*-test). Data are shown as mean and SD of three biological replicates (**F**). Data are representative of three biological replicates (**A–H**).

To achieve rapid intracellular degradation of SARS-CoV-2 NP, we sought to find ways to diminish USP22 levels. Interestingly, although SARS-CoV-2 is a virus that typically warrants antiviral therapy, such as the use of Ritonavir or Remdesivir, etc (23), a significant number of COVID-19 patients have received antibiotic treatment regardless of whether they had a bacterial infection or not (24). Antibiotic treatment has proven to be beneficial when used rationally (25). Therefore, we examined several commonly used clinical antibiotics to determine if any could affect USP22. Surprisingly, we found that the antibiotic cefotaxime sulbactam significantly reduced USP22 levels (Fig. 5A). Next, we confirmed that cefotaxime sulbactam could reduce SARS-CoV-2 NP levels by regulating USP22 in a dose-dependent manner (Fig. 5B). Since cefotaxime and sulbactam are often used in combination therapy for better therapeutic outcomes, we aimed to identify the component responsible for the observed effect. The results indicated that cefotaxime had no effect on USP22 (Fig. 5C), while sulbactam effectively downregulated USP22 in both dose- (Fig. 5D) and time-dependent manner (Fig. 5E). Furthermore, cells transfected with Flag-NP and treated with sulbactam inhibited decreased SARS-CoV-2 NP levels, which correlated with reduced USP22 levels (Fig. 5F). In addition, we also confirmed that sulbactam downregulated USP22 (Fig. 5G) and consequently affected SARS-CoV-2 NP levels in A549 cells (Fig. 5H). Meanwhile, CCK8 experiments showed that at different concentrations (0, 2, 4, and 8 mg/mL), sulbactam did not exhibit significant cytotoxicity against HEK293T cells after 12 h of treatment (Fig. 5I), suggesting that they have minimal toxicity to normal cells. In contrast, when the concentration of sulbactam reached 12 mg/mL, the drug exhibited cytotoxicity. In addition, the concentrations we used in the cell experiments were much lower than the concentrations of antibiotics used in the clinic intravenously. Altogether, these findings indicated that sulbactam is capable of reducing USP22 and contributing to the downregulation of SARS-CoV-2 NP (26).

### Sulbactam administration reduces USP22 levels and downregulates SARS-CoV-2 NP *in vivo*

**Fig 6:**
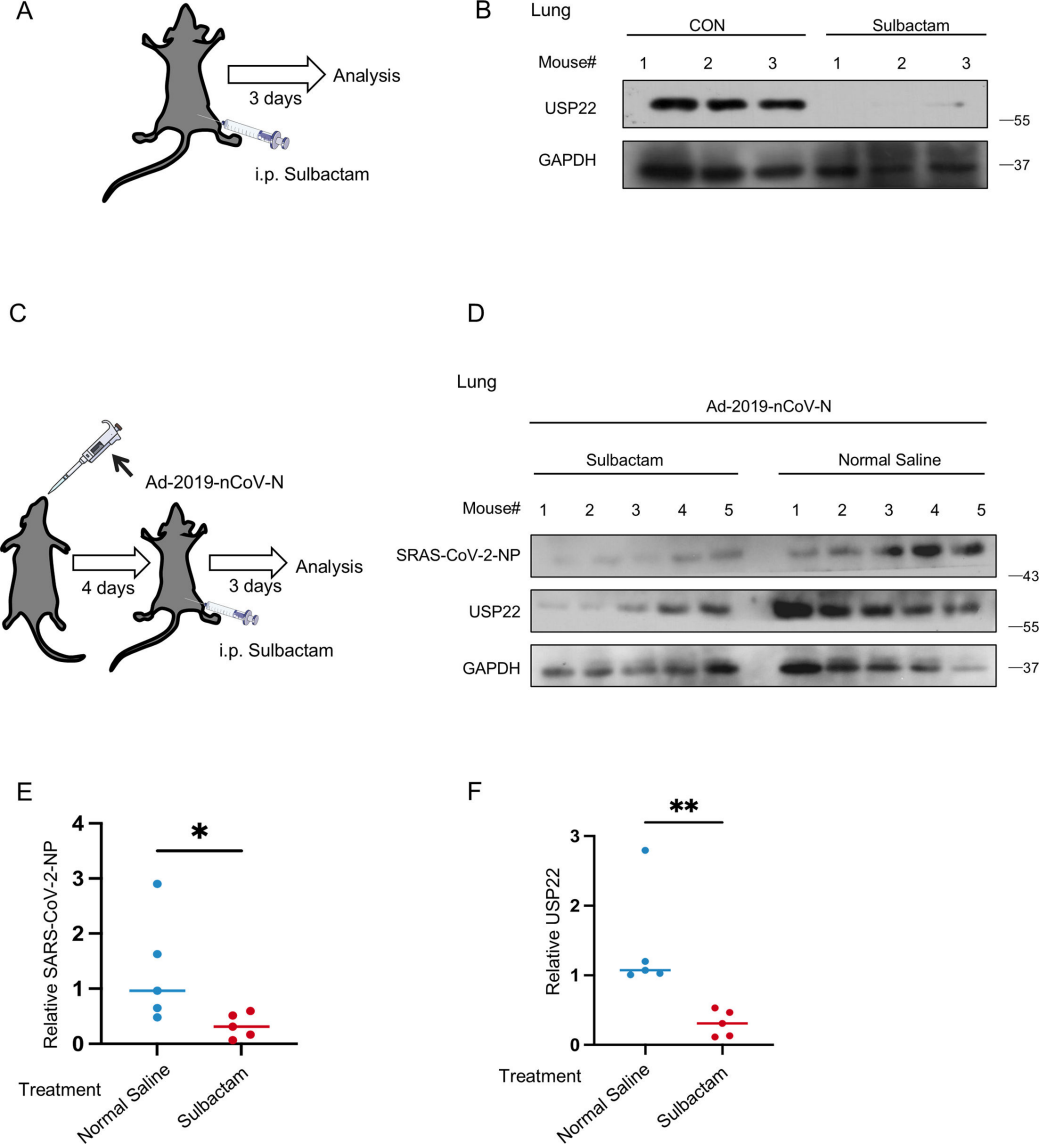
Sulbactam administration reduces USP22 levels and downregulates SARS-CoV-2 NP *in vivo.* (**A**) The mice were administered with sulbactam (100 mg/kg, i.p.) once daily for 3 days. (**B**) Western blot analysis of USP22 in the lung tissues from the mice administered as (**A**). (**C**) The mice were anesthetized via isoflurane inhalation and then intranasally dosed with 1 × 10^11^ PFU per gram body Ad-2019-nCoV-N. After 4 days, mice were administered with sulbactam (100 mg/kg, i.p.) once daily for 3 days. (**D**) Western blot analysis of USP22 and SARS-CoV-2 NP in the lung tissues from the mice treated as (**C**). (**E**) The striped images of SARS-CoV-2 NP from (**D**) were imported into Image **J**. The pixel count of all the strips was measured and then calculated in GraphPad Prism software. (**F**) The striped images of USP22 from (**D**) were imported into Image J. The pixel count of all the strips was measured and then calculated in GraphPad Prism software. *P < 0.05 and **P < 0.01 (two-tailed unpaired Student’s *t*-test). Data are representative of three biological replicates (**B and D**).

We further sought to observe how ss sulbactam administration affects USP22 and SARS-CoV-2 NP levels *in vivo*. Following the antibiotic administration protocol described in mouse experiments (27), we administered ss sulbactam intraperitoneally for 33 consecutive days (Fig. 6A). The results showed that ss sulbactam significantly reduced USP22 protein levels in the lung tissues (Fig. 6B). As we all know, recombinant adenovirus vectors have been widely used in experiments (28) and vaccine development (29). Therefore, we utilized a modified adenovirus expressing SARS-CoV-2 NP (Ad-2019-nCoV-N). Previous reports indicated that adult BALB/c mice intranasally dosed with 2 × 10^6^ PFU/mL of the virus exhibit significant pulmonary infection (30). Based on our cell line studies, we adjusted the infection concentration of Ad-2019nCoV-N to 1 × 10^11^ PFU per gram of body weight. Mice under anesthesia were first intranasally dosed with Ad-2019-nCoV-N and,, after 4 days, were administered with sulbactam or normalsaline as described (Fig. 6C). By immunoblotting for SARS-CoV-2 NP and USP22 in lung tissues, we observed a clear decrease in SARS-CoV-2 NP and USP22 protein levels in mice treated with ss sulbactam (Fig. 6D). Next, we analyzed the grayscale images of SARS-CoV-2 NP protein levels from Fig. 6D. Results showed that mice treated with ss sulbactam expressed lower SARS-CoV-2 NP (Fig. 6E). Moreover, grayscale analysis of USP22 protein levels from Fig. 6D confirmed that ss sulbactam downregulates USP22 levels *in vivo* (Fig. 6F).

## DISCUSSION

After infecting cells, viruses exploit the cellular environment to enhance pathogenicity and survival, employing various host cell mechanisms such as transcription, translation, and PTMs, while evading the host immune response (31, 32). Identifying host cell proteins that interact with viral proteins is crucial for understanding the immune escape mechanisms of viruses and providing effective therapeutic targets (32). Similar to other coronaviruses, SARS-CoV-2 enters the cell via its S protein, which binds to ACE2 (33), making the S protein the most extensively studied (34, 35). However, the N protein is highly conserved across coronavirus species and is one of the most abundantly expressed structural proteins during viral infection (3). SARS-CoV-2 NP is also associated with inflammation and organ dysfunction. Studies have linked it to heightened inflammatory response, lung injury, and kidney damage following SARS-CoV-2 infection (36, 37), making SARS-CoV-2 NP a predictor of disease severity (38). Therefore, we urgently want to know how NP protein uses the host cell mechanism to complete the immune escape, leading to extensive virus replications.

Here, we provide evidence that SARS-CoV-2 NP is degraded by the ubiquitin-proteasome pathway in cells, a process regulated by deubiquitinating enzymes. Furthermore, by immunoprecipitation, we revealed the interaction between USP22 and SARS-CoV-2 NP. Moreover, the deubiquitinase USP22 significantly reduces the K63-linked ubiquitination of SARS-CoV-2 NP, thereby decreasing the degradation of SARS-CoV-2 NP. In eukaryotes, K48-linked ubiquitination primarily mediates protein degradation through the proteasomal pathway, while K63-linked ubiquitin chains play key roles in processes such as DNA damage repair, cell signaling, and autophagy (39). Although there are no reports of other coronaviruses mediating degradation through K63-linked ubiquitination, studies have shown that K63-linked ubiquitination modifications can facilitate protein degradation via the proteasomal pathway (40). In this study, we demonstrated that USP22 can remove the K63-linked ubiquitination of SARS-CoV-2 NP but not the K48-linked ubiquitination by transfecting exogenous ubiquitination plasmids of different types and performing detection with endogenous antibodies. This, in turn, reduces the degradation of SARS-CoV-2 NP through the proteasomal pathway. However, whether the roles of K63 and K48 ubiquitination modifications in the ubiquitin-proteasome pathway are identical remains to be further explored.

*Usp22* mRNA is expressed in omnifarious regions, especially in alveolar cells of the lung (21). Similarly, the USP22 protein is expressed in multiple tissue cells *in vivo*, making it readily available for SARS-CoV-2 NP to utilize. Most of what has been published about SARS-CoV-2 NP and ubiquitination modification has focused on how SARS-CoV-2 NP affects cellular antiviral signaling pathways. For instance, SARS-CoV-2 NP inhibits MAVS ubiquitination, leading to the suppression of IFN production (41). Additionally, SARS-CoV-2 NP interacts with RIG-I protein to suppress the IFN response (42). However, these studies primarily describe how SARS-CoV-2 NP accomplishes immune escape by inhibiting the interferon signaling pathway. Our study reveals a more direct and clearer mechanism, where SARS-CoV-2 NP degrades less and achieves enhanced intracellular stability through the deubiquitination activity of USP22.

Additionally, we were even more surprised to find that sulbactam significantly downregulates USP22, thereby accelerating SARS-CoV-2 NP degradation. As a β-lactamase inhibitor, sulbactam is typically used in antibacterial therapy. It seems like it has absolutely nothing to do with antiviral activity. However, for the first time, we have uncovered its antiviral mechanism. There is considerable debate regarding the use of antibiotics during SARS-CoV-2 infections. Some hold the opinion that COVID-19 has a relatively low rate of bacterial co-infection but a high rate of antibiotic use (43), which could have complex long-term effects on antimicrobial resistance (44). Conversely, other studies suggest that combining antibiotic treatment can reduce COVID-19-associated mortality (45). Notably, our study provides the first evidence that sulbactam, traditionally an antimicrobial drug, can also exert antiviral effects. Existing medicines are already widely used in the clinic with favorable safety and pharmacokinetic profiles and can be rapidly put into practical use. That is why the pharmacological exploitation of already existing drugs is very important and valuable. Our study provides an important basis for drug repurposing and encourages the exploration of more potent properties of other existing medicines. However, rational use of antibiotics remains essential and must be strictly adhered to.

Due to the limitation in obtaining the SARS-CoV-2, we had to use a modified adenovirus expressing the SARS-CoV-2 NP for animal experiments. Additionally, although we have demonstrated that sulbactam can downregulate USP22, we have not yet elucidated the detailed mechanisms through which sulbactam affects USP22. Thus, additional cellular and animal experiments will be conducted to investigate the mechanism of USP22 downregulation by Sulbactam.

In summary, our results offer new insights into the ubiquitination of SARS-CoV-2 NP and the immune escape mechanism of SARS-CoV-2. Moreover, we revealed that certain antibiotics may possess antiviral properties, providing new perspectives on the early use of antibiotics in viral infections.

## MATERIALS AND METHODS

### Mice

C57BL/6 mice were all male and were obtained from the Laboratory Animal Center of Soochow University and housed in a specific-pathogen-free environment within the Experimental Animal Center’s Animal Facility at the same institution. Mice aged 6–8 weeks were utilized for all experiments.

### Cell culture and reagents

HEK293T and A549 cells were obtained from the American Type Culture Collection. All cells were mycoplasma negative and were maintained at 37°C in an atmosphere containing 5% CO_2_. HEK293T and A549 cells were grown in Dulbecco’s modified Eagles’s minimal essential medium (DMEM) (HyClone) supplemented with 10% fetal bovine serum (FBS) (GIBCO, Life Technologies), 100 µg/mL streptomycin, and 100 units/mL penicillin. Lung tissues were harvested from male mice aged 6–8 weeks.

### Plasmids and transfection

The Flag-NP plasmid was a gift from Dr. Jianfeng Dai (Soochow University, China). Myc-NP and Flag-HA-USP22 mutant (C185S) were generated using PCR amplified from Flag-NP and Flag-HA-USP22. Flag-HA(FH)-tagged human DUBs, including Flag-HA-USP22, were gifts from Dr. J. Wade Harper (Harvard Medical School, Addgene Plasmids). HA‐Ub (K6, K11, K27, K29, K33, K48, and K63) was kindly provided by Dr. Lingqiang Zhang (State Key Laboratory of Proteomics, China). All plasmids were verified through sequencing.

All mutations were generated by the QuickChange Lightning site-directed Mutagenesis Kit (TIANGEN, KM101). All transient transfections were carried out using LongTrans (Ucallm) according to the manufacturer’s instructions.

### Western blot

Cells were lysed using the lysis buffer composed of 1% Nonidet P-40 (NP-40), 150 mM NaCl, 20 mM Tris-HCl (pH 7.4), 0.5 mM EDTA, and 50 µg/mL phenylmethylsulfonyl fluoride (PMSF). Equal amounts of total proteins were subjected to SDS-PAGE and subsequently transferred onto polyvinylidene fluoride membranes (Millipore). The membranes were then blocked for 30 minutes at room temperature using either 5% skim milk or 5% bovine serum albumin. After blocking, the membranes were incubated with the appropriate primary antibodies, followed by horseradish peroxidase-conjugated goat anti-mouse or anti-rabbit secondary antibodies (Bioworld). Visualization of the membranes was performed using ECL Prime (Thermo Scientific) after three washes with phosphate-buffered saline with Tween 20.

The antibodies with the indicated dilutions were as follows: anti-USP22 (Abcam, ab195289, 1:3,000), anti-HA (Abcam, ab9110, 1:3,000), anti‐Flag (Sigma, F7425, 1:5,000), anti‐Myc (Abmart, M20002H, 1:3,000), anti-GAPDH (Proteintech, 60004–1-lg, 1:3,000), anti-Ubiquitin (Santa Cruz, sc-66180, 1:1,000), anti‐K48 Ub (CST, 4289S, 1:1,000), anti-K63 Ub (CST, 5621S, 1:3,000), and anti-SARS-CoV-2 NP (ABclonal, A20142, 1:2,000).

### Immunoprecipitation

Cells were lysed as mentioned above. Then cell lysates were incubated with specific antibodies on a rotor at 4°C, followed by the addition of Protein Gagarose beads (Millipore) that had been washed twice. The mixture was then incubated on a rotor for 2–3 h at 4°C. After three washes with a buffer containing 150 mM NaCl, the immunoprecipitates were analyzed by western blot. To normalize the IP, the target proteins were first immunoprecipitated, diluted in a loading buffer, and then analyzed by immunoblotting. Based on the immunoblotting results, equal amounts of immunoprecipitated target proteins were used for interaction or ubiquitination analysis. Whole-cell lysates (30 µg) were used as an input control.

### Real-time quantitative PCR

Total RNAs from various cell types were extracted by using the TRIzol reagent (Invitrogen). RT-qPCR was conducted using SYBR Green (Selleck) on a StepOne Plus real-time PCR system (Applied Bioscience). Relative gene expression levels were calculated using the 2^−ΔΔCt^ method. All target gene quantifications were normalized to the control gene β-actin, and data are expressed as fold changes relative to either unstimulated or uninfected cells.

The results represent the mean ± SD from three independent experiments. The primer sequences are as follows: for SARS-CoV-2 N, forward primer 5′-AAGCTGGACTTCCCTATGGTG-3′ and reverse primer 5′-CGATTGCAGCATTGTTAGCAGG-3′; for β-actin, forward primer 5′-ACCAACTGGGACGACATGGAGAAA-3′ and reverse primer 5′-ATAGCACAGCCTGGATAGCAACG-3′.

### CRISPR-Cas9-mediated genome editing

The lenti-CRISPRv2 vector was generously provided by Dr. Fangfang Zhou (Soochow University, China). To achieve gene KO, small guide RNAs were initially cloned into the lenti-CRISPRv2 vector, which was then transfected into cells. Forty-eight hours post-transfection, the cells were subjected to puromycin selection (1.5 µg/mL) for 3 days. The effectiveness of the knockout was confirmed through immunoblotting analysis. Subsequently, the cells were used for further experimentation. The guide RNA for human *Usp22*: 5′-GCAACCCGCCTGTGAAGAT-3′.

### Cell cytotoxicity assay

HEK283T cells were seeded into a 96-well plate at a density of 1 × 10^4^ cells per well and incubated in a water-saturated incubator with 5% CO_2_, with four replicates per group at least. When the cell density reached approximately 70%, on a clean bench, the lid of the well plate was opened, and different concentrations of sterilized sulbactam were added to treat the cells. Cells were returned to a 37°C incubator. After 12 h, the culture medium was then removed, and 100 µL of 10% CCK8 solution was added to each well. The plates were further incubated for 1.5 h in the incubator, and the OD values of each well were measured at a wavelength of 450 nm to calculate cell viability. The experiment was performed in triplicate.

### Viral infection *in vivo*

We synthesized the Adenovirus-2019-nCoV-N from WZ Biosciences and confirmed its ability to infect the mouse lungs and cause the expression of SARS-CoV-2 NP in the lung tissues by western blot. *In vivo* viral infection experiments, 8-week-old mice were randomly assigned to two groups. Mice were first anesthetized and intranasally dosed with 1 × 10^11^ PFU per gram body Ad-2019-nCoV-N. Four days after infection, sulbactam (100 mg/kg) was intraperitoneally injected into mice. After 72 h, mouse lung tissues were collected. Western blot was conducted to analyze SARS-CoV-2 NP and USP22 protein levels.

### Statistical analysis

A two-tailed unpaired Student’s *t*-test was used to assess the significance between groups. Differences were considered statistically significant when *P* < 0.05. In the figures, *P*-values are represented by asterisks as follows: *P < 0.05, **P < 0.01, and ***P < 0.001.

## Data Availability

No large primary data sets were generated or deposited in external repositories.
